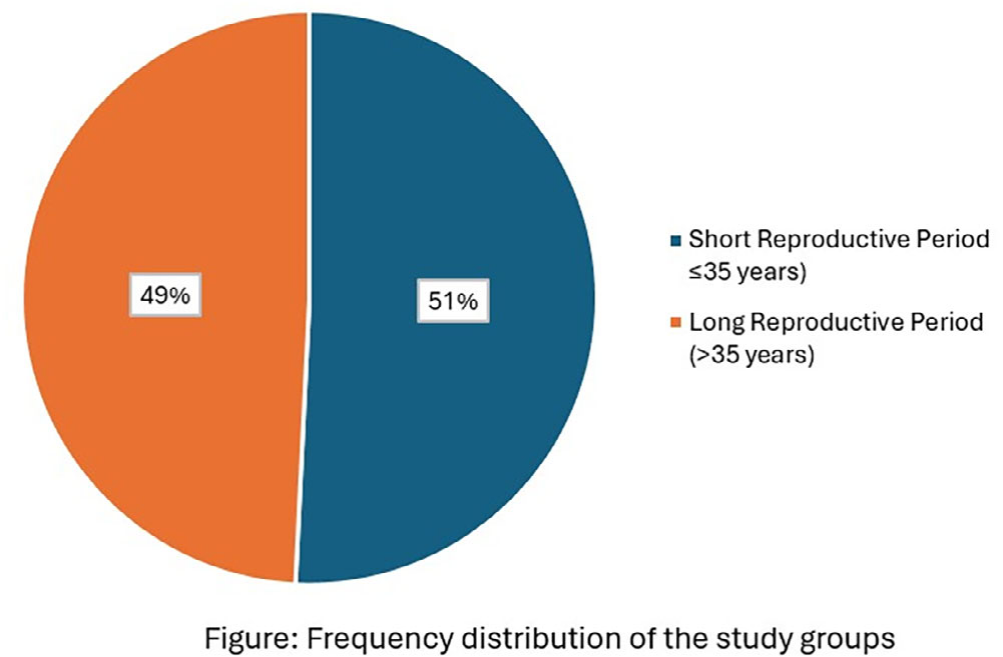# Reproductive period and brain health in post‐menopausal women from urban India

**DOI:** 10.1002/alz70860_104720

**Published:** 2025-12-23

**Authors:** Aishwarya Ghosh, Monisha S, Thomas Gregor Issac

**Affiliations:** ^1^ Centre for Brain Research, Indian Institute of Science, Bangalore, Karnataka, India; ^2^ Centre for Brain Research, Indian Institute of Science, Bengaluru, Karnataka, India

## Abstract

**Background:**

A longer Reproductive Period (RP), or a late onset of menopause is associated with better cognitive performance and reduced risk of age‐related brain atrophy in later life. In this context, our study aims to explore the cognitive abilities and brain volume differences among post‐menopausal women in an urban Indian city, specifically comparing those with shorter and longer reproductive periods.

**Method:**

Post‐menopausal women (*N* = 583) aged ≥45 years were recruited in the current study from the baseline cohort of the Tata Longitudinal Study of Aging. The participants underwent detailed clinical, cognitive, biochemical and neuroimaging (3T MRI) assessments. The RP was calculated by subtracting the age at menarche from the age at menopause for all the participants. The participants were categorised into two groups based on the median RP of 35 years—≤35 years (short RP) and >35 years (long RP) [Figure]. T‐test/Mann‐Whitney U test was used to determine group differences. Generalised Linear Models (GLM) were employed to explore the association between RP and cognitive functions and brain volumes.

**Result:**

The mean age of the participants was 62.12±8.1 years with no significant difference between the groups. The short RP group had significantly less years of education (*p* <0.001), a lower age at first childbirth (*p* = 0.043), and low levels of folic acid (*p* = 0.04) as compared to the long RP group. GLM adjusted for age, education, age at first childbirth, and folic acid levels revealed that the short RP group had poorer attention function (β = ─0.417, *p* = 0.023). The short RP group also had lesser total grey matter (β = ─6464.403, *p* = 0.026), cortex (β = ─4827.836, *p* = 0.044), and right hemisphere cortical (β = ─2971.033, *p* = 0.018) volumes as suggested by GLM adjusted for age, education, and total intracranial volumes.

**Conclusion:**

Our study found that a longer reproductive period is associated with better attention function, preserved grey matter, and larger cortical volumes in post‐menopausal women. These findings highlight the neuroprotective effects of extended estrogen exposure, potentially reducing the risk of age‐related brain atrophy. Future research should investigate the mechanisms underlying these associations and their public health implications.